# Hoarseness Due to Aortic Arch Aneurysms

**DOI:** 10.21470/1678-9741-2019-0352

**Published:** 2020

**Authors:** Shi-Min Yuan

**Affiliations:** 1 The First Hospital of Putian, Teaching Hospital, Fujian Medical University, Putian, People’s Republic of China.

**Keywords:** Hoarseness, Aneurysm, Infected, Recurrent Laryngeal Nerve, Conservative Treatment, Trachea, Aorta Aneurysm

## Abstract

**Objective:**

To give an overview of the Ortner’s syndrome caused by an aortic arch aneurysm.

**Methods:**

By comprehensive retrieval of the pertinent literature published in the past two decades, 75 reports including 86 patients were collected and recruited into this study along with a recent case of our own.

**Results:**

The aortic arch aneurysms causing hoarseness were most commonly mycotic aneurysms. In this patient setting, in addition to the left recurrent laryngeal nerve, trachea was the most commonly affected structure by the aortic arch aneurysm. Surgical/interventional/hybrid treatments led to a hoarseness-relieving rate of 64.3%, much higher than that of patients receiving conservative treatment. However, hoarseness recovery took longer time in the surgically treated patients than in the interventionally treated patients.

**Conclusion:**

The surgical and interventional treatments offered similar hoarseness-relieving effects. Surgical or interventional treatment is warranted in such patients for both treatment of arch aneurysms and relief of hoarseness.

**Table:** 

Abbreviations, acronyms & symbols	
CT	= Computed tomography
MeSH	= Medical Subject Headings
PRISMA	= Preferred Reporting Items for Systematic Reviews and Meta-analyses

## INTRODUCTION

In 1897, Ortner described a series of three cases of mitral stenosis who were also suffering from hoarseness of voice because of left recurrent laryngeal nerve palsy, and it was then termed as Ortner’s syndrome^[1]^. This condition is rare, and its incidence is difficult to ascertain. The cardiovascular etiologies of hoarseness can be congenital, valvular, aortic, or supra-aortic vascular disorders^[2]^. Aortic aneurysm of any etiology can be a risk factor leading to cardiovascular hoarseness, whereas thoracic aortic aneurysms represent only 5% of the cases^[2]^. Nevertheless, Ortner’s syndrome caused by an aortic arch aneurysm is an even rarer entity, and its clinical features, treatments of choice, and patients’ outcomes are unknown although sporadic cases are continuously reported. The purpose of this study is to give an overview of the Ortner’s syndrome caused by an aortic arch aneurysm.

## METHODS

The Preferred Reporting Items for Systematic Reviews and Meta-analyses (PRISMA) statement guidelines were followed for completing the present meta-analysis. Publications from 2000 to the present time were comprehensively searched in the PubMed database. The Medical Subject Headings (MeSH) terms and keywords that were applied for the literature retrieval included “hoarseness”, “dysphonia”, “aphonia”, “Ortner’s syndrome”, “cardiovocal syndrome”, “left recurrent laryngeal nerve palsy”, “left vocal cord palsy”, “aneurysm/pseudoaneurysm”, and “aortic arch”. The screening of the bibliographic references helped to complete the literature retrieval. One hundred and twenty-seven articles were found related to the topic and keywords in the literature search, and 75 articles, which met the inclusion criteria during preliminary assessment, were included in the review. The exclusion criteria were articles reporting: hoarseness due to (pseudo)aneurysm of other segments of aorta (n=12), aortic arch/ascending aorta dissection (n=8), ductal aneurysm (n=3), aneurysms of the left/right/aberrant right subclavian artery (n=3), cardiomegaly (n=3), right-sided aortic arch without/with Kommerell’s diverticulum (n=3), cervical aortic arch (n=1), and pulmonary hypertension (n=1); hoarseness due to aortic arch aneurysm with no patient information available (n=8); aortic arch aneurysm while patients did not present with hoarseness (n=5); aortic arch repair techniques (n=3); thoracic aorta trauma (n=1); and coarctation of the aorta (n=1).

The data independently extracted from each study were the study population; demographics; clinical symptoms; associated disorders; the size, shape, dimension, extension, location, and nature of the arch aneurysms; treatment of choice; hoarseness recovery; and patients' outcomes.

The IBM SPSS Statistics 22.0 software was used for statistical analysis. The measurement data were expressed in mean±standard deviation and were compared by independent sample/paired *t*-test. The categorical variables were compared by Fisher’s exact test. The predictive risk factors for compression of other adjacent organs/tissues were assessed by multinomial logistic regression. *P*<0.05 was considered statistically significant.

## RESULTS

**Fig. 1:**
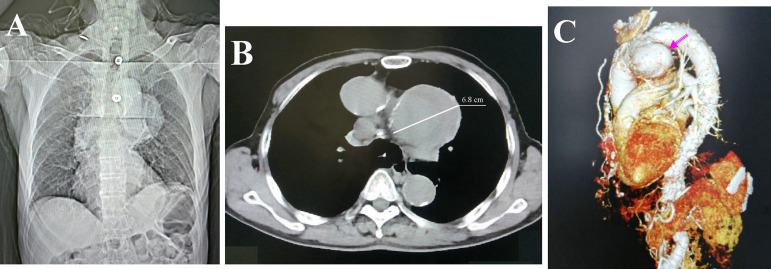
Computed tomography (CT) of an aortic arch pseudoaneurysm in a 73-year-old male patient presenting with a two-week hoarseness. (A) The first frame imaging of CT scan showed a protruding aortic arch into the left upper thoracic cavity; (B) an axial view of the aortic arch pseudoaneurysm measuring 6.8 cm in diameter; and (C) the aortic arch pseudoaneurysm arising from the inferior aspect of the arch on three-dimensional CT.

In total, 86 patients were included in the 75 articles^[1-75]^. We recently had a 73-year-old male patient, who presented with two-week hoarseness with laryngoscopic evidence of left vocal cord paralysis. Chest computed tomography showed a giant aortic arch pseudoaneurysm (diameter 6.8 cm) with mural thrombosis (Figure 1). Altogether, 87 patients were recruited into this study.

Patients were at the age of 67.1±13.8 (range: 28-89; median: 70) years (n=80). There were 69 (79.3%) male and 18 (20.7%) female patients with an absolute male gender predominance (χ^[2]^=59.8, *P*<0.001). There was no age difference between the male and female patients (67.1±13.9 years *vs*. 70.7±9.4 years, *P*=0.346).

Hoarseness was a sole symptom in 32 (36.8%) patients^[1,9,11,12,18,21,22,31-34,36,41,43,45,47,50,54-56,58,59,62,63,65-68,74,75]^, a major symptom in 34 (39.1%) patients^[2,5,6,8,10,13-16,19,24,28,31,32,38,39,44,46,49,51-53,57,61,64,70]^, and an accompanying symptom in 21 (24.1%) patients^[3,4,7,17,20,23,25-27,29,30,32,35,37,40,42,48,60,69,72,73]^ (χ^[2]^=5.1, *P*=0.079).

Except for hoarseness, 45 (51.7%) patients had 76 other symptoms (Table 1). The duration of hoarseness before current admission was 5.6±11.7 (range: -0.07-72; median: 2) months (n=55) (time of hoarseness that occurred after admission was recorded as a minus).

**Table 1:** Other symptoms of patients with Ortner's syndrome.

Symptom	n (%)
Dyspnea/breathlessness/shortness of breath^[7,8,13,14,25,26,30,35,49,51,52,60,72,64]^	14 (18.7)
Cough^[2,16,17,24,27,35,42,46,48,49,51,61,64,73]^	14 (18.7)
Chest/back pain^[2-4,10,13,15,19,23,31,32,40,69,70]^	13 (17.3)
Dysphagia^[3,13,16,19,20,38,42,73]^	8 (10.7)
Fever/fever of unknown origin^[17,29,37,40]^	4 (5.3)
Weight loss^[7,20,37,49]^	4 (5.3)
Hemosputum/hemoptysis^[29,53,48]^	3 (4.0)
Fatigue^[32,60]^	2 (2.7)
Poor appetite^[2,20]^	2 (2.7)
Weakness^[2,37]^	2 (2.7)
Choking^[39]^	1 (1.3)
Aspiration^[32]^	1 (1.3)
Nasal obstruction^[5]^	1 (1.3)
Sore throat^[5]^	1 (1.3)
Sputum^[31]^	1 (1.3)
Dizziness^[57]^	1 (1.3)
Headache^[57]^	1 (1.3)
Orthopnea^[52]^	1 (1.3)
Claudication pain in the legs^[52]^	1 (1.3)
Shock^[44]^	1 (1.3)

The arch aneurysms were purely arch-affected in 76 (87.4%) patients^[1,2,4,6-18,20-49,52-56,58-61,63-66,68-71,73,74]^, the aneurysm was extending from the ascending aorta to the aortic arch in four (4.6%) patients^[3,31,57,62]^, from the aortic arch to the descending aorta in three (3.4%) patients^[51,67,75]^, and there were arch-thoracoabdominal aortic aneurysm^[19]^, aneurysm extending from the ascending aorta to the descending aorta^[72]^, arch and left subclavian artery aneurysms^[50]^, and an aortic arch aneurysm with a proximal descending aorta aneurysm^[5]^ in one (1.1%) patient, each.

In 27 (31.0%) patients, the exact locations of the arch aneurysm were described. Most of the aneurysms affect the distal portion of the aortic arch (Table 2).

**Table 2:** Locations of the arch aneurysms.

Location	n (%)
Distal^[6,17,18,29,31,32,56,75]^	11 (40.7)
Inferior/basal^[4,12,13,16,33,35,55,64,71]^	9 (33.3)
Anterior^[14,53]^	1 (3.7)
Inferoposterior^[11]^	1 (3.7)
Inferolateral^[59]^	1 (3.7)
Inferolateral & anterior^[63]^	1 (3.7)
Lateral^[7,68]^	1 (3.7)
Posterior^[9]^	1 (3.7)
Proximal^[5]^	1 (3.7)

The etiology of the aneurysmal formation was described in 28 (32.2%) patients, with mycotic arch aneurysm being a major etiology (Table 3).

**Table 3:** Etiology of aortic arch aneurysms.

Etiology	n (%)
Mycotic^[6,22,28,29,32,37,38,40]^	12 (42.9)
*Salmonella*^[15,29]^	2 (16.7)
*Clostridium septicum*^[38]^	1 (8.3)
*Clostridium septicum^,^ Enterobacter^,^ and Streptococcu*s^[40]^	1 (8.3)
*Pseudomonas aeruginosa*^[32]^	1 (8.3)
*Streptococcus dysgalactiae*^[32]^	1 (8.3)
*Candida albicans*^[32]^	1 (8.3)
Unspecified^[6,22,28,32,37]^	5 (41.7)
Atherosclerotic^[12,20,28,31]^	11 (39.3)
Penetrating aortic ulceration^[4,18,33]^	3 (10.7)
Traumatic^[43]^	1 (3.6)
Atherosclerotic/traumatic^[34]^	1 (3.6)

The mean maximal diameter of arch aneurysms was 6.8±2.7 (range: 3-15; median: 6.4) cm (n=55). The shapes of arch aneurysms were described for 47 patients: 36 (76.6%) aneurysms were saccular nine (19.1%) were fusiform^[27,42,44,51,54,57,61,62,72]^, one (2.1%) was bilobed^[73]^, and one (2.1%) was irregularly shaped^[38]^. A mural thrombus was present in 34 (20.7%) patients^[1,2,7,8,11-14,18,19,22,29,31,34,38,39,42,45,46,48-52,54-56,60,62,63,68-70]^. In 12 (13.8%) patients, the arch aneurysms were pseudoaneurysms^[4,17,26,33,36,41,43,46,48,55^^],73]^. Left vocal cord paralysis was evidenced in 39 (44.8%) patients: by laryngoscopic examination in 34 (87.2%) patients^[2-5,7-9,11,12,14,18,20,21,23-26,33-39,41,42,45-47,52,56-59,62]-64,[66,68,71,73-75]^, by nasopharyngoscopy in three (7.7%) patients^[55,69,70]^, and by bronchoscopy in two (5.1%) patients^[48,54]^. Nevertheless, one patient had right vocal cord paralysis instead^[67]^.

Apart from compression of the left/right recurrent laryngeal nerve by the aortic arch aneurysm, 13 (14.9%) patients had compressions of other adjacent organs/tissues. The trachea was the most commonly affected organ (Table 4).

**Table 4:** Other 21 adjacent organs/tissues compressed by aortic arch aneurysms in 13 patients.

Adjacent organs/tissues	n (%)
Trachea^[8,13,20,23,26,72]^	6 (30)
Esophagus^[3,13,20]^	3 (15)
Main pulmonary artery^[14,23,31]^	3 (15)
Left pulmonary arteries^[14,53]^	2 (10)
Right ventricular outflow tract^[53]^	1 (5)
Origin of left carotid & left subclavian arteries^[20]^	1 (5)
Right bronchus^[27,67]^	1 (5)
Right hilum^[3]^	1 (5)
Right phrenic nerve^[3]^	1 (5)
Superior lobe of the left lung^[5]^	1 (5)
Superior vena cava^[3]^	1 (5)

In 17 (19.5%) patients, there was an associated disorder, including arch aneurysm rupture (n=5)^[3,30,44,48,69]^ (one was impending rupture^[69]^), aortoesophageal fistula (n=2)^[40,48]^, aortopulmonary fistula (n=2)^[17,30]^, aortic dissection (n=2)^[21,75]^, hemidiaphragmatic paralysis (n=2)^[2,52]^, descending aorta pseudoaneurysm (n=1)^[43]^, iliopsoas muscle abscess with a history of left common iliac artery aneurysm (n=1)^[32]^, left hemothorax (n=1)^[72]^, and pericardial and pleural effusions (n=1)^[73]^.

The predictive risk factors for compressions of other adjacent organs/tissues were assessed by admitting the size (*P*=0.059), shape (*P*=0.712), mural thrombus (*P*=0.410), and false aneurysm (*P*=0.999) as dependent variables by multinomial logistic regression. Only arch aneurysmal size was a quasi-determinant for the compressions.

The treatment of choice was not described in 18 patients. Thirteen patients refused to receive a surgical/interventional treatment^[3,7,14,19,20,22,23,35,39,52,59,60,64]^. Two patients died suddenly before the treatment started^[69,73]^. One patient was advised to consult the surgeon for possible surgical treatment^[49]^. Two patients were on a follow-up^[66,67]^. Nine patients chose conservative treatment due to their poor conditions that could not support a surgical treatment^[2,11,44,47,54,61,71,72,74]^. Of the remaining 42 patients, 19 (19/42, 45.2%) patients underwent a surgical treatment (an arch replacement in 16 [16/19, 84.2%] patients^[5,10,12,15,21,27,29-32,38,48,65]^, patch reconstruction of the aorta in two [2/19, 10.5%] patients^[13,17]^, and the operation method was not indicated in one [1/19, 5.3%] patient^[57]^), 18 patients (18/42, 42.9%) received an endovascular aortic repair by a stent graft insertion^[6,26,28,33,34,40-42,56,58,75]^, and five (5/42, 11.9%) patients were treated with hybrid procedures, including stent graft plus woven Dacron vascular graft in two patients^[50,53]^, and endovascular frozen elephant trunk replacement of the ascending aorta and aortic arch^[70]^, left carotid-subclavian bypass plus stent graft^[4]^, and open-arch repair and descending aorta stent grafting^[43]^ in one patient, each.

The surgical approach for open arch replacement was described for eight patients: via a median sternotomy in six (6/8, 75%) patients^[25,29,30,38,53,70]^ and via a left lateral thoracotomy in two (2/8, 25%) patients^[5,12]^.

Cardiopulmonary bypass technique was described for 10 patients: partial cardiopulmonary bypass with no heart arrest in one (10%) patient^[13]^, full cardiopulmonary bypass in four (40%) patients^[5,12,50,70]^, and deep hypothermic circulatory arrest with cerebral perfusion in five (50%) patients^[15,25,29,30,38]^.

In 32 patients, the hoarseness-relieving effect was reported: the hoarseness was relieved in 12 (37.5%) patients, improved in seven (21.9%) patients (improved by injection of hyaluronic acid for voice in one patient^[74]^), and persisted in 13 (40.6%) patients (χ^[2]^=2.9, *P*=0.234). The total effective rate was 59.4% (19/32). The hoarseness recovery, improvement, and persistent rates did not differ between the surgical and interventional treatment groups (Table 5). However, the total effective rate of the patients receiving surgical/interventional/hybrid treatments was much higher than that of patients with conservative treatment (64.3% [18/28] *vs.* 0% [0/4], χ^[2]^=5.9, *P*=0.028). Time for hoarseness relief was much shorter in the interventionally treated patients than in the open surgically treated patients (Table 6).

**Table 5:** Hoarseness-relieving effects with different treatments of aortic arch aneurysm.

Treatment	Recovered	Improved	Persisted
Surgical (n=10)	4 (37.5)	3 (37.5)	3 (25)
Interventional (n=16)	7 (38.5)	3 (15.4)	6 (46.2)
Hybrid (n=2)	1 (50)		1 (50)
Conservative (n=4)			4 (100)
χ^2^ (surgical *vs*. interventional)	0.0	0.4	0.2
*P*-value (surgical *vs*. interventional)	1.000	0.644	1.000

**Table 6:** Hoarseness-relieving time after surgical/interventional/hybrid treatment (months).

Treatment	Recovered	Improved	Persisted
Surgical (n=8)	18.0±8.5	1	20.3±9.0
Interventional (n=13)	3.6±2.8	0.3, 3	1
Hybrid (n=1)	3	--	--
*t*-value (surgical *vs*. interventional)	3.217	--	--
*P*-value (surgical *vs*. interventional)	0.015	--	--

Patients were on a follow-up of 8.3±7.2 months (n=25). Patients’ outcomes were known for 59 patients: 36 (61.0%) patients recovered, one (1.7%) patient improved, four (6.8%) patients were complicated and eventually recovered after treatment, nine (15.3%) patients’ conditions did not change, and nine (15.3%) patients died. The total effective rates showed a significant difference between the four treatment groups (91.3% *vs*. 75% *vs*. 100% *vs*. 7.7%, χ^[2]^=30.3, *P*<0.001), whereas the mortality rates did not differ (4.3% *vs*. 6.3% *vs*. 0% *vs*. 28.6%, χ^[2]^=6.3, *P*=0.096) (Table 7).

**Table 7:** Patients' outcomes subjected to different treatments.

Treatment	Recovered	Improved	Complicated	Unchanged	Died
Surgical (n=23)	21 (91.3)		1 (4.3)		1 (4.3)
Interventional (n=16)	12 (75)		3 (18.8)		1 (6.3)
Hybrid (n=3)	3 (100)				
Conservative (n=14)		1 (7.1)		9 (64.3)	4 (28.6)
Total (n=56)	36 (64.3)	1 (1.8)	4 (7.1)	9 (16.1)	6 (10.7)

## DISCUSSION

The left recurrent laryngeal nerve arises from the left vagus nerve at the level of the aortic arch curve, and then it curves around the aorta on the outer side of the ligamentum arteriosum ascending along the tracheoesophageal groove. This prolonged course makes it vulnerable to injury by the lesions of the surrounding structures^[54]^.

Hoarseness of voice is a frequent presentation of otolaryngology diseases due to a neoplastic, surgical, idiopathic, traumatic, central, or infectious etiology. Nevertheless, cardiovascular hoarseness, especially hoarseness due to an aortic arch aneurysm, is very rare. The bulging cardiovascular structures can frequently compress the left recurrent laryngeal nerve leading to left vocal cord paralysis, rendering patients presenting with hoarseness^[2]^.

In addition to hoarseness, the common symptoms of patients with Ortner’s syndrome were dyspnea and dysphagia^[72,73]^. However, in this patient setting with an arch aneurysm as an etiology, the common symptoms were dyspnea, cough, and chest pain.

The etiology of thoracic aortic aneurysm can be typically categorized as heritable or degenerative. In the cohort of patients with Ortner’s syndrome due to an aortic aneurysm, the underlying etiologies can be degenerative, traumatic, dissecting, and atherosclerotic, all pathological changes of the aorta that create compression on the left recurrent laryngeal nerve. In this report, mycotic infection prevailed the underlying causes of the aortic arch aneurysms, followed by an atherosclerotic etiology.

The aortic arch aneurysms lead not only to compression of the left recurrent laryngeal nerve, but also compression of other adjacent organs/tissues in one-fifth of the Ortner’s syndrome patients, with the trachea being the most affected organ. Moreover, the arch aneurysmal size was found to be a quasi-determinant leading to the adjacent organ compression as disclosed by the present study.

Indirect laryngoscopy becomes a more common technique for the diagnosis of vocal cord palsy. Further investigations include echocardiography, computed tomography, and magnetic resonance imaging. Early diagnosis of Ortner’s syndrome is essential for starting timely treatment, restoring vocal cord function, and avoiding permanent damage of the patients.

The treatment of choice of aortic arch aneurysms can be an open surgery or an interventional procedure. Usually, the conventional treatment of mycotic aneurysms is open aneurysmectomy, along with the debridement of the adjacent infectious tissues. *In situ* tube graft insertion or extra-anatomic bypass grafting may be at risk of infection, and thus with less promising long-term outcomes^[6]^. In spite of advanced diagnostic modalities and refined interventional therapies of today, the mortality rate of surgical treatment of mycotic aneurysm remains high. Endovascular grafts have been widely used for the treatment of aortic aneurysms as a very appealing alternative to open aortic surgery, in particular for the patients in whom surgical procedures carry high risks^[6]^. The increasingly sophisticated interventional technology has enabled the treatment of lesions of the critical segments of the aorta, such as the thoracic and arch levels^[6]^.

The recovery in hoarseness is highly variable. In a comprehensive review including 58 patients with Ortner’s syndrome, the hoarseness resolved in 44.8%, improved in 29.3%, persisted in 22.4%, and exacerbated in 3.5% of patients. The present study based on a cohort of patients with aortic arch aneurysm revealed a bit lower recovery and improving rates, but higher persisted rate of hoarseness. This might be explained by the fact that long-term compression of the left recurrent laryngeal nerve by an aortic aneurysm may take longer time to recover.

Morales et al.^[28]^ described that aneurysm size increase but no endoleak was responsible for the persistent hoarseness after endovascular therapy of the aortic aneurysms, whereas aortic aneurysm size decrease was not necessarily associated with hoarseness relief after the procedure^[28]^. The present study illustrated that the surgical and interventional therapies offered similar hoarseness-relieving effects; and time for hoarseness relief was much shorter in the interventionally treated patients than in the surgically treated patients. It hinted that interventional therapy might be a treatment of choice that could lead to a higher hoarseness-relieving rate.

## CONCLUSION

Aortic arch aneurysms may cause compression of the left recurrent laryngeal nerve and other adjacent organs/tissues as well. The overall hoarseness-relieving (including recovery and improvement) rate of patients receiving non-conservative treatments was 64.3%, much higher than those with conservative treatment. However, hoarseness recovery took longer time in the surgically treated patients than in the interventionally treated patients. Surgical or interventional treatment is warranted in such patients for the treatment of arch aneurysms and for hoarseness recovery as well.saccular^[1,2,5-7,9,10,13,14,16,18,20,22,25,29,31,35,37,39,45,47,49,50,52,55,56,58,59,63-65,70,71,74,75]^, nine (19.1%) were fusiform^[27,42,44,51,54,57,61,62,72]^, one (2.1%) was bilobed^[73]^, and one (2.1%) was irregularly shaped^[38]^. A mural thrombus was present in 34 (20.7%) patients^[1,2,7,8,11-14,18,19,22,29,31,34,38,39,42,45,46,48-52,54-56,60,62,63,68-70]^. In 12 (13.8%) patients, the arch aneurysms were pseudoaneurysms^[4,17,26,33,36,41,43,46,48,55,73]^.

**Table:** 

Author's roles & responsibilities	
SMY	Substantial contributions to the conception or design of the work; acquisition, analysis, and interpretation of data for the work; drafting the work and revising it critically for important intellectual content; agreement to be accountable for all aspects of the work in ensuring that questions related to the accuracy or integrity of any part of the work are appropriately investigated and resolved; final approval of the version to be published
